# Supplementary material for: Automated eye-tracking for Parkinson's disease diagnosis: a proof-of-concept cascade classifier study
Source: Clin Park Relat Disord. 2026 Oct 2;15:100509. doi: 10.1016/j.prdoa.2026.100509 (PMC13661203; doi:10.1016/j.prdoa.2026.100509)
Supplement: Supplementary file 1 [file mmc1.pdf]

# SCHEMATIC NETWORK DIAGRAM

## Oculomotor Circuits Affected in Parkinson's Disease

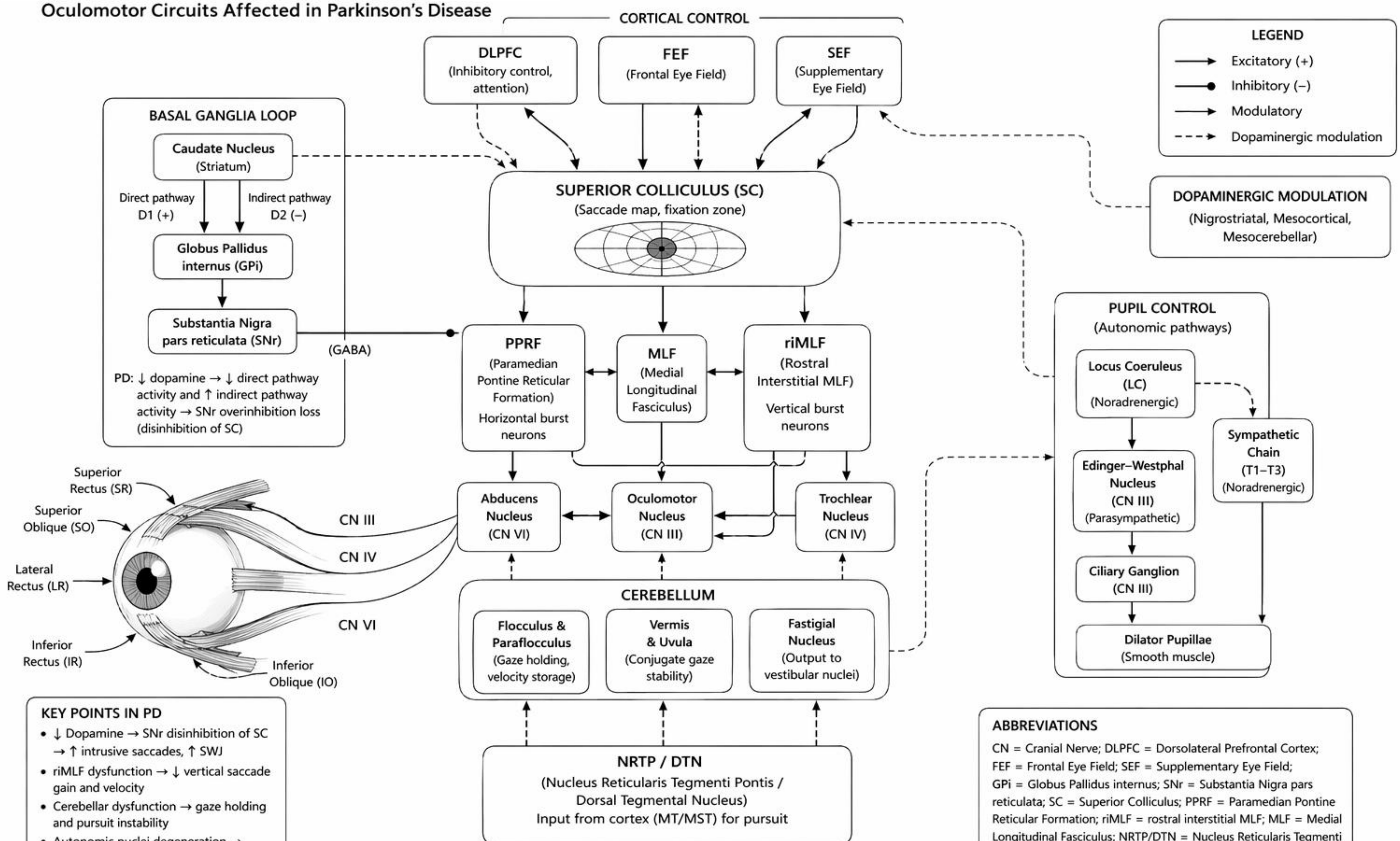


### KEY POINTS IN PD

- ↓ Dopamine → SNr disinhibition of SC → ↑ intrusive saccades, ↑ SWJ
- riMLF dysfunction → ↓ vertical saccade gain and velocity
- Cerebellar dysfunction → gaze holding and pursuit instability
- Autonomic nuclei degeneration → pupillary dysfunction

### ABBREVIATIONS

CN = Cranial Nerve; DLPFC = Dorsolateral Prefrontal Cortex; FEF = Frontal Eye Field; SEF = Supplementary Eye Field; GPi = Globus Pallidus internus; SNr = Substantia Nigra pars reticulata; SC = Superior Colliculus; PPRF = Paramedian Pontine Reticular Formation; riMLF = rostral interstitial MLF; MLF = Medial Longitudinal Fasciculus; NRTP/DTN = Nucleus Reticularis Tegmenti Pontis / Dorsal Tegmental Nucleus.
